# Global Gridded Emission Inventory of Organophosphate Flame Retardants from 2010 to 2020

**DOI:** 10.1021/acs.est.4c06504

**Published:** 2024-09-09

**Authors:** Haibo Ma, Chao Wang, Huabing Suo, Yandi Huang, Yuanhui Huo, Gang Yang, Yu Yan, Tao Huang, Hong Gao, Jianmin Ma, Zhiyong Xie

**Affiliations:** † Key Laboratory for Environmental Pollution Prediction and Control, Gansu Province, College of Earth and Environmental Sciences, Lanzhou University, Lanzhou 730000, P. R. China; ‡ Laboratory for Earth Surface Processes, College of Urban and Environmental Sciences, Peking University, Beijing 100871, P. R. China; § Helmholtz-Zentrum Hereon, Institute of Coastal Environmental Chemistry, Geesthacht 21502, Germany

**Keywords:** organophosphate flame retardants, emission inventory, atmospheric transport model, validation

## Abstract

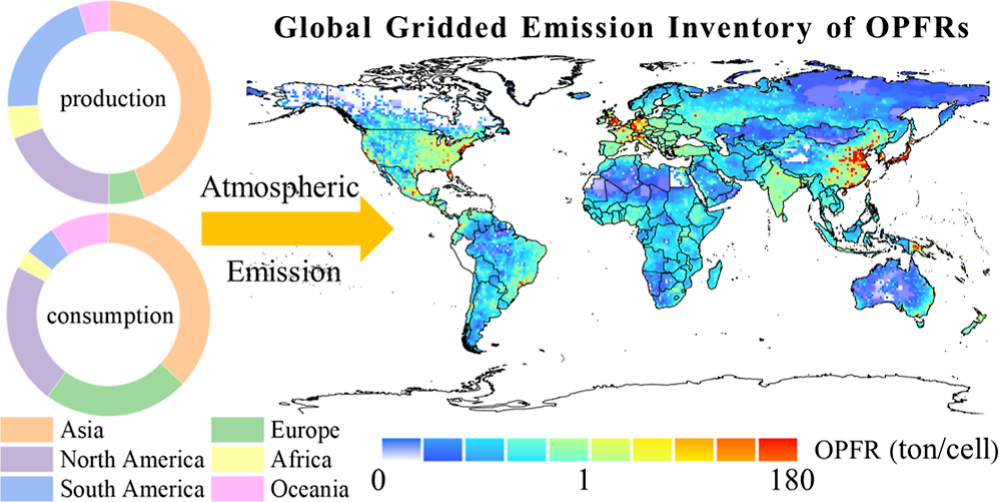

As a substitute for brominated flame retardants, organophosphate flame retardants (OPFRs) have become a global concern due to their high toxicity and bioaccumulation. To paint an overall picture of OPFRs in the global environment, the present study develops a gridded global emission inventory of OPFRs on a spatial resolution of 1 × 1° from 2010 to 2020. Revealing a 3.31% average annual increase in emissions, totaling 21,324.42 tons. The production process is the primary source, accounting for 55.43% of emissions, with consumption processes making up the rest. Major sources are in Asia, North America, and Europe. The inventory is verified by implementing emission data into a global atmospheric transport model to predict OPFR concentrations in the global environment and comparing modeled concentrations with field sampled data. The results indicate that the inventory is reliable except for the pristine polar region, where the emission inventory and modeled concentrations underestimate OPFR levels in the atmosphere, likely resulting from ignorance of chemical reactions and the secondary derivative of parent OPFRs during their global long-distance atmospheric transport in the model. This comprehensive data set aids in formulating OPFR emission control policies and assessing health risks.

## Abstract 2

This study has created a global gridded emission inventory for OPFRs, demonstrating the distribution of global OPFR emissions and the current state of pollution.

## 1 Introduction

Brominated flame retardants (BFRs) are commonly utilized globally for fire protection purposes,^1^ yet concerns have been raised regarding their toxicity, bioaccumulation, and environmental persistence. As a result, many BFRs have been included in the Stockholm Convention for elimination.^2^ As an alternative, organophosphate flame retardants (OPFRs) have attained wide use as a substitute for BFRs,^3^ leading to a surge in their production, which now accounts for approximately 30% of the total global flame retardants.^8^ From 2010 to 2020, the total emissions from production and consumption processes worldwide have risen from 1648.37 to 2239.37 tons.^4^ Recent studies have indicated that OPFRs exhibit detrimental effects on the environment and human health that are not inferior to those assigned persistent organic pollutants (POPs) assigned to the “Stockholm Convention”. The adverse biological and human health effects of various congeners of OPFRs have been demonstrated in both in vivo and in vitro studies. Notably, it has been identified that these chemicals can apply non-negligible toxicity at concentrations above health thresholds.^5^ The accumulation of OPFRs in the human body has been linked to a range of negative health outcomes in numerous epidemiological studies, including endocrine disruption, carcinogenicity, neurotoxicity, reduced birth weight, developmental toxicity, and reproductive toxicity.^6,7^

As key components of organophosphate esters (OPEs),^8^ OPFRs are often incorporated into various types of materials including textiles, rubber, plastics, and wood. In comparison with covalent bonds, OPFR binds to the products noncovalently and tends to exhibit high volatility or semivolatility. This can lead to leaching, loss, and release of the flame retardant into the environment during each phase of the product’s lifespan, including production, use, disposal, and recycling.^9,10^ The production and consumption rates of OPFR have been growing annually. In 2010, the global production and consumption volumes of OPFRs were 364,300 tons and 374,700 tons, respectively. By 2020, these figures had risen to 504,300 tons and 472,900 tons, respectively,^4^ causing increasing OPFR pollution in the environment worldwide. In 2006, Castro-Jiménez et al.^11^ reported high Σ_14_OPFR concentrations in the atmosphere around the Black Sea, ranging from 400 to 6000 pg/m^3^. In a field study across the Great Lakes between 2007 and 2013, the atmospheric levels of Σ_14_OPFRs were as high as 100–2340 pg/m^3^.^3^ Rauert et al.^12^ collected Σ_18_OPFRs air samples from 48 cities in the world and found concentration range of 69–7770 pg/m^3^. Möller et al.^13^ conducted cruising measurements of OPFR in the atmosphere along two shipping routes from East Asia to the North and South Poles between 2010 and 2011. They reported eight types of OPFR concentrations ranging from 19 to 2000 pg/m^3^. These results revealed worldwide contamination of OPFR in the environment. Even though these field campaigns produced useful and precise OPFR pollution status in different locations, a comprehensive understanding of global OPFRs on a high spatiotemporal resolution is essential. There is an urgent need to construct a global emission inventory of OPFR, which will provide fundamental information on OPFR to the scientific community and policy makers to assess their environmental and health consequences and to develop effective emission reduction strategies and implement rigorous pollution control measures. However, to the best of our knowledge, such a emission inventory, either on a national scale or high spatially resolved scale, is not available. It is imperative to underscore that the gridded atmospheric emission inventory serves as a fundamental component in the understanding of source-receptor relationships and provides indispensable input data for computational models to simulate the local, regional, and global environmental fate, cycling, and exposure risk of OPFRs. The primary objective of this study is to (1) compile and analyze production and utilization data of OPFRs from 2010 to 2020 globally and develop a high-resolution gridded atmospheric emission inventory; (2) assess, in a concise fashion, the impact of different sectors on OPFR emissions and levels of pollution and employ the Canadian Model for Environmental Transport of Organochlorine Pesticides (CanMETOP) model to simulate and delineate the spatial distribution patterns of global OPFR pollution in the atmosphere and seawater.

## 2 Materials and Methods

### 2.1 National-Level OPFR Productions and Emissions

In 2021, there were 367 companies across the globe involved in the production of 56 types of OPFR monomers and 62 types of OPFR mixtures. More than 200 of these companies were located in eastern China.^26^ The consumers of flame retardants were mainly identified in North America, Western Europe, Japan, and other parts of Asia (China, India, and South Korea), accounting for 34, 29, 10, and 27% of the OPFR consumption, respectively.^14−17^ The global production and consumption data for OPFR are sourced from an industry report purchased from the Beijing Zhongshang Hua Information Technology Research Institute, titled "Global and China Organophosphate Flame Retardants Industry Research and Prospects Jing Dynamic Analysis Report 2021 Edition (2010–2020)″ (http://www.zsjjyjy.com/report/406538.html). Due to considerable uncertainties in global production and usage volumes, we also collected relevant data from literature and available statistic yearbooks in different countries.^6,13,17−27^ Upon scrutiny and verification of these data, we recalibrated some of the contents in the industry reports and obtained more comprehensive national-level production and consumption data of OPFR. We compared the collected production and consumption data with industry-wide reports to identify any potential inconsistencies. A key step to examine the inconsistencies of data sources is to compare modeled concentrations driven by the emission inventory derived from the background data in the production and consumption sectors with field measured concentrations data. The discrepancies between modeled and sampled mean concentrations can help discern the potential errors in the data sources in different countries and regions and correct the data uncertainties, as will be elaborated below.

The methodology for the quantification of OPFR emissions was based on a bottom-up approach and emission factors for different sectors. Given the broad spectrum of OPFR and their extensive utilization as additives, emissions were generated during both the production and consumption stages, respectively. Based on the literature, the OPFR consumption of plastics, rubber, textiles, coatings, paper, and wood contributed 70, 20, 5, 3, 1, and 1% to the total OPFR consumption, respectively.^17^ The use of OPFR is often featured by complex mixtures, and the manner and proportion of their mixing can lead to considerable uncertainty in their emission factors. Therefore, we exclusively focused on comprehensive emission factors under different usage methods, specifically the emissions from the six consumption processes mentioned above, of which OPFR utilized as additives in plastic manufacturing were considered the primary source of emissions during the consumption process. As per the Organization for Economic Co-operation and Development (OECD), the emissions of OPFR in the plastic additive industry encompass four processes: (1) raw material processing, (2) mixing, (3) conversion, and (4) molding. The emissions from the production process are relatively manageable and are divided into three stages: raw material processing, synthesis, and conversion. Given the potential of OPFRs’ risk to human health and the ecological environment, we have opted for relatively higher emission factors.^28−30^ The emission factor method pertains to the process wherein a predetermined portion of OPFR is emitted into the atmosphere during both production and consumption stages.^17^ As presented in Table S1, the emission factor in the production process is 0.255%, and in the consumption processes, it ranges from rubber (0.1%) to paper (0.26%). The detailed emission factors for different sectors are listed in Table S1 of the Supporting Information. The national OPFR emissions from the six consumption processes are defined as follows
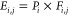
1where *E*_*i*,*j*_ is the atmospheric emission of the OPFR in production and consumption sector *i* (*i* = 1, 2, ..., 7) and phase *j* (*j* = 1, 2, 3), (unit: 10,000ton);

*P*_*i*_ is the production and consumption amount of OPFR (unit: %). The production and consumption data for various countries are provided in Supporting Information 1; *F*_*i*,*j*_ is the atmospheric emission factor of the OPFR in production and consumption sector *i* (*i* = 1, 2, ..., 7) and phase *j* (*j* = 1, 2, 3) (unit: 10,000ton).

### 2.2 Emission Gridding

To capture local and regional variations in emissions, identify emission hotspots, and develop targeted strategies for emission reduction, one needs allocating emissions from a national total to specific geographic locations, such as grid cells. This process is essential for air quality modeling as well. To do so, we need to select suitable spatially resolved proxy data sets to allocate national regional-scale emissions to self-defined grid cells across the globe. Considering that anthropogenic commercial activities are the main processes for OPFR emissions,^23^ the selection of suitable spatial data sets should incorporate data pertaining to human activities.^13,25,31,32^ To allocate the consumption processes of plastic, rubber, textiles, coatings, paper, and wood into spatially resolved grids or locations, we introduced gridded population density data with the resolution of 1° × 1° latitude and longitude (Socioeconomic Data and Applications Center, SEDAC) as a proxy data set. The emissions from the production process are directly correlated to the national industrial capacity. Therefore, we utilized the Gross Domestic Product (GDP) of the secondary industry (GDP_2_) as a proxy for emission gridding in the production process. Given the scarcity of gridded GDP_2_ data, following Huang et al.,^43^ we adopted satellite remote sensing and observed high spatial resolution global nighttime light (NTL) to estimate gridded GDP_2_ because industrial activities, especially manufacturing and mining, often involve significant energy consumption and nighttime operations, leading to a strong correlation between NTL intensity and economic output.^33−35^ The NTL index at a resolution of 1° × 1° latitude and longitude is collected from the Earth Observation Group, as shown in Figure S1. The linear relationship between the nighttime light and GDP_2_ is given by
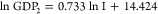
2where GDP_2_ represents the GDP data of the secondary industry (in units of 10,000 CNY/km^2^);

*I* is the nighttime light index with a resolution of 1° × 1° latitude and longitude.

### 2.3 Atmospheric Transport Model

In this study, the CanMETOP is adopted to simulate the concentrations of OPFRs in the global atmosphere and seawater.^36^ Detailed descriptions of the CanMETOP can be referred to previous studies.^36−38^ CanMETOP was developed to simulate atmospheric transport, the environmental fate and cycling in multiple compartments, and the source-receptor relationships of persistent toxic chemicals. The model encompasses three-dimensional atmospheric transport, turbulent diffusion, dry and wet deposition, air–soil and air–water exchange, and degradation in various environmental matrices. The air–soil and air–water modules of CanMETOP consider diffusive gas deposition from the air to the surface medium (soil and water) and volatilization from the surface medium to the air. The model has a horizontal resolution of 1° × 1° latitude and longitude and a temporal resolution of 20 min. Under a terrain-following coordinate (39), CanMETOP has 14 vertical levels (0, 1.5, 3.9, 10, 350, 700, 1200, 2000, 3000, 5000, 7000, 9000, and 11,000 m). The model’s capabilities and performance have been validated through numerous field studies and model evaluations, demonstrating its capability in POP modeling.^17,37,38,40^ The meteorological data used in the model, such as wind, atmospheric pressure, temperature, and precipitation, are sourced from the 1° × 1° latitude/longitude 6 h objective analysis data and the Final Global Operational Analysis provided by the National Centers for Environmental Prediction (NCEP, http://dss.ucar.edu/datasets/ds083.2/). The geographical data include terrain elevation, land use, soil organic carbon, and surface roughness length. The terrain elevation and land use data are obtained from the Oak Ridge National Laboratory (http://daac.ornl.gov/cgi-bin/catalog.pl?l). To investigate the environmental behavior of OPFR in the ocean, this study has added a water–sediment module to the CanMETOP model, which employs a mass balance model developed by Mackay et al.^41^ Detailed descriptions of the CanMETOP model can be found in Text S1 of Supporting Information. To objectively evaluate the performance of CanMETOP in representing ΣOPFRs, a group of 12 predominant OPFR congeners that have been detected in various environments were selected,^22,42^ including triethyl phosphate (TEP), tripropyl phosphate (TPP), tri-iso-butyl phosphate (TIBP), tri-*n*-butyl phosphate (TNBP), tris(2-chloroethyl) phosphate (TCEP), tris(2-chloroisopropyl) phosphate (TCPP), tris(2-butoxyethyl) phosphate(TBEP), tris(1,3-dichloroisopropyl) phosphate (TDCPP), triphenyl phosphate (TPP), 2-ethylhexyl diphenyl phosphate (ethylhexyl diphenyl phosphate), tris(2-ethylhexyl) phosphate (TEHP), and tris(methylphenyl) phosphate (TMPP). The molecular formulas and abbreviations of these OPFR congeners are also documented in Table S2, along with their respective physicochemical parameters. The average physicochemical parameters of the Σ_12_OPFRs are presented in Table S3. Figure S2 is a flowchart illustrating the framework of data collection, gridded emission inventory development, and verification.

### 2.4 Uncertainties

The primary sources of uncertainty influencing the output of the CanMETOP modeled concentrations, including the emission inventory and physicochemical properties of OPFRs, are examined. Since the CanMETOP model considers complex dynamics and physical processes, such as dry and wet deposition, precipitation scavenging, horizontal and vertical advection, and turbulent diffusion, a first-order error propagation method is employed to assess the CanMETOP‘s sensitivity and uncertainty.^37^ This approach allowed us to estimate the 95% confidence intervals for global atmospheric and seawater concentrations simulated by CanMETOP, which signifies lower uncertainty. More detailed descriptions of sensitivity and uncertainty analysis and adjusted relevant parameters are presented in Text S2 and Table S4 of Supporting Information.

To estimate the uncertainties of the global OPFR emission inventories developed in this study, rigorous Monte Carlo simulations^38,43^ were performed to scrutinize potential variations in the output of the OPFR emissions herein. The primary sources of uncertainty in this research were identified as population, economic status, and emission factors. These data were collected from the World Bank’s database, and we ascribe a relatively low degree of uncertainty to them. Our primary focus was on the emission sources and emission factors. The coefficients of variation (CV) pertaining to proportions of OPFR usage were adopted from Jiang et al.,^39^ while respective CV for different emission factors were prespecified within the range of 19–44%. The CV for several emission sector categories and emission factors is presented in Table S5. Each input parameter was considered to follow a normal distribution, serving as a surrogate for randomness. We repeat Monte Carlo simulations for 10,000 times at a 95% confidence level to generate uncertainty information for emissions from different sources. The results are depicted in Figure S3 and feature a normal distribution.

## 3 Results and Discussion

### 3.1 Global OPFR Emissions

**Figure 1:**
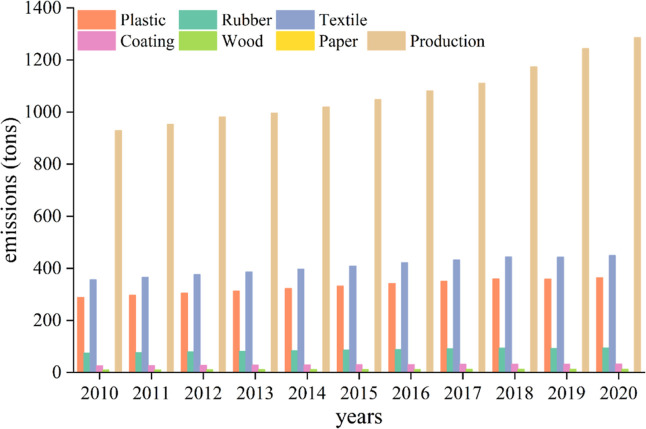
Annual total OPFR emissions from consumption and production process in seven major sectors (tons) from 2010 to 2020 in the globe.

As mentioned before, OPFRs are a complex group of prevalent additives utilized in a range of industrial sectors, including plastics, textiles, rubber, and wood. The emissions associated with these production processes are directly proportional to the level of industrial development within a nation; as a result, emissions are positively correlated with the country’s economic status and population size. Over the 11 year period from 2010 to 2020, our result indicates that 21,324.42 tons of OPFRs were emitted into the atmosphere with an annual growth rate of 3.31%. Figure 1 provides a comprehensive overview of the annual atmospheric emissions from seven key sectors from 2010 to 2020. Among these sectors, emissions from the production process accounted for 55.43% of the total, followed by textiles (20.99%), plastics (17.02%), rubber (4.42%), coatings (1.52%), and wood and paper (0.6%). The growing emissions from all sectors varied year by year. Table S6 provides the digital data.

**Figure 2:**
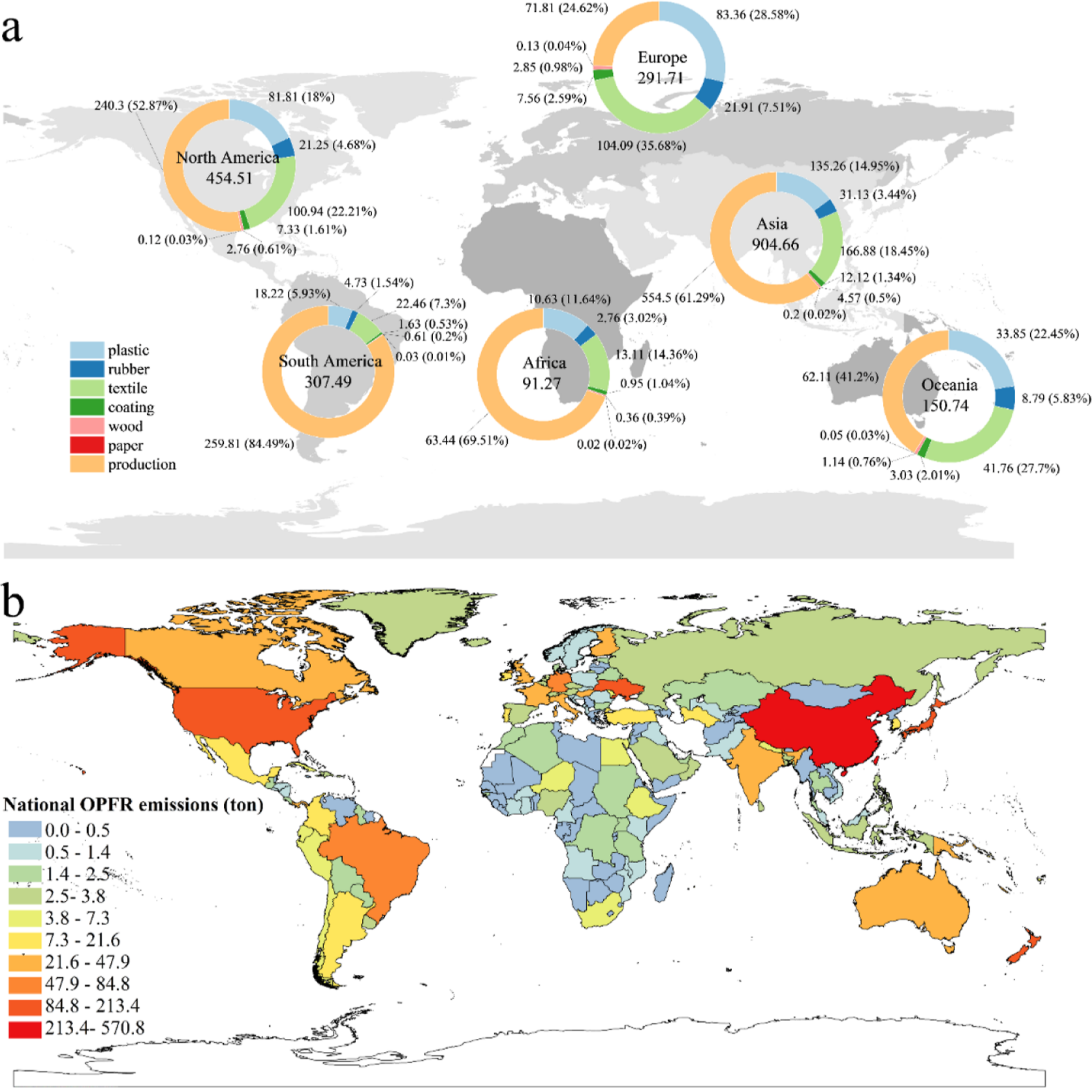
Global OPFR emissions in 2020. (a) Continental-level emissions; pie charts represent the total continental emissions and proportions from various sectors. (b) National-level emissions across the globe.

Figure 2a illustrates continental total OPFR emissions in 2020 across the globe and the proportion of OPFR emissions from different sectors in the continental total. Total global emission in 2020 was estimated to be 2200.41 tons. The highest proportion of emissions from the production process was discerned in South America, the region with a relatively low population but a relatively developed industry. The highest proportion of emissions from the consumption was observed in Europe, attributable primarily to the relocation of its high OPFR emission industries to Asia and North America and the Europe’s stringent emission mitigation policies implemented.^44^ As a result, the majority of OPFR emissions in Europe was sourced from the consumption, instead of the production. The emissions from large-scale production account for the largest proportion of the total emissions in all continents, except for Europe, where the consumption sectors of plastics, textiles, and rubber industries contributed the most to the emission from the consumer sector, primarily attributed to the frequent use of OPFR containing products on a daily basis and the higher emission factors in consumer products. Among seven continents, Asia released 904.66 tons, or 41.11% of the global total, to the atmosphere, followed by North America at 454.51 tons (20.66%), South America at 307.49 tons (13.97%), Europe at 291.71 tons (13.26%), Oceania at 150.74 tons (6.85%), and Africa at 91.27 tons (4.15%), respectively. The results identify China and the United States (US) as major contributors to the continental total emissions in Asian and North American continents due to their larger sources in the OPFR-rich products^23^ associated with their large populations and industrial activities. Figure 2b shows the total emissions in each country in 2020. Higher OPFR emitters in 2020 can be identified in China, America, Japan, Brazil, Germany, France, India, and the United Kingdom. These countries all together contributed 74.76% of the global total atmospheric emissions of OPFRs. These countries share the common characteristics of having developed industries and large populations. We also examined the rest of the 100 countries with low population and less industrial activities. The result indicates that these countries collectively contributed only 1.79% to the total global emission of OPFRs.

### 3.2 Gridded Global Emission Mapping

**Figure 3:**
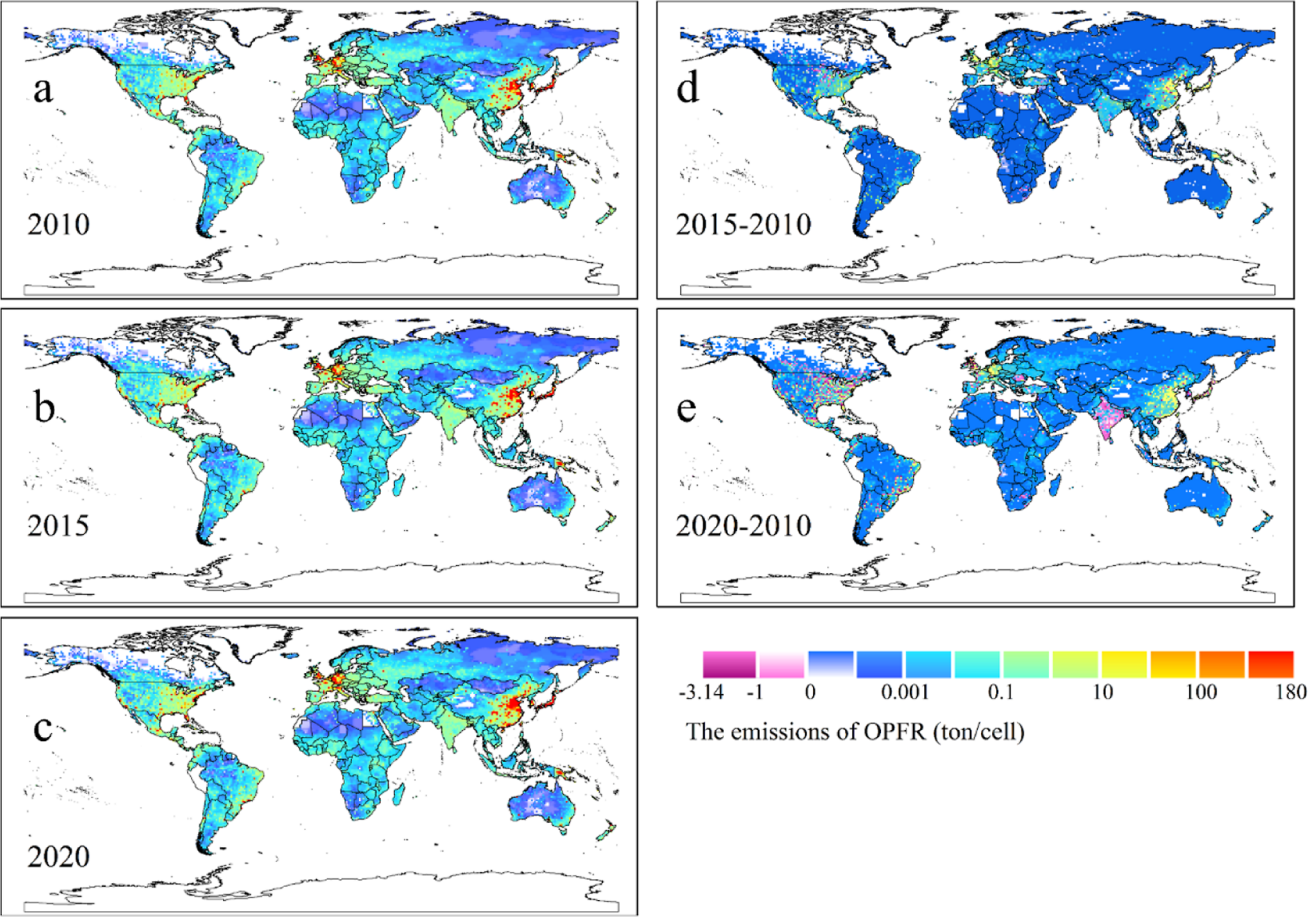
Global gridded OPFR emission inventories on a 1° × 1° (longitude/latitude) spacing (unit: ton/cell). (a) Gridded emission inventory in 2010; (b) gridded emission inventory in 2015; (c) gridded emission inventory in 2020; (d) difference in gridded emissions between 2015 and 2010; and (e) difference in gridded emissions between 2020 and 2010.

The global gridded OPFR emission distribution in selected years is depicted in Figure 3, which is consistent with the national-level emissions, as shown in Figure 2b from a spatial perspective. Comparisons of global emission distributions in 2010 (Figure 3a), 2015 (Figure 3b), and 2020 (Figure 3c) show insignificant changes in spatial distributions of OPFR emissions for the ten years, which also suggests that the major sources of OPFRs did not alter markedly. Overall, from 2010 to 2020, the spatial distribution pattern of atmospheric emissions has remained largely consistent, with high concentrations in those countries having high populations and well-developed industries such as China, Japan, the United Kingdom, France, Germany, Italy, and the US. The emissions in Asia, North America, South America, and Europe were primarily sourced from eastern China and Japan, the eastern US, Brazil, and eastern European countries, respectively. The global average emission per land grid cell was 0.119 tons/cell in 2010, which escalated to 0.139 tons/cell in 2015, and further increased to 0.163 tons/cell in 2020. As illustrated in Figure 3d,e, from 2010 to 2015, OPFR emissions increased by 221.84 tons and by 547.35 tons from 2010 to 2020. Growing emissions occurred primarily in eastern China, India, Western Europe, and some countries in South America. In contrast, those regions with slow economic growth and industrial development, such as Africa, have not experienced considerable changes in emissions over the 11 year period. There are also areas with declining emissions as shown by negative emissions differences, such as central China, parts of India, and some regions in the eastern US due to population growth and industry relocation. Although OPFR production and consumption continue to rise, OPFR emissions at some grid cells show declining trends with a varying degree, characterized by negative emission differences (Figure 3d,e), such as those grid cells in India, eastern Europe, and the border regions between the US and Canada.

Compared with the national-level emissions, the gridded emission distribution provides a more precise and comprehensive view for localizing and interpreting emission globally. The intensive emissions primarily occur in the most industrialized, urbanized, and densely populated regions, where flame retardants are extensively produced and utilized. As such, the gridded distribution can help formulate more precise decisions for emission mitigation strategies and provide substantial data support to computer simulations pertaining to the analysis of concentrations and source-receptor relationships.

### 3.3 Global Environmental Fate Modeling and Emission Verification

To verify the reliability and accuracy of the OPFR emission inventory, we implemented OPFR emissions into the CanMETOP to (1) simulate the global atmospheric OPFR fate after its release to the atmosphere and (2) compare modeled OPFR concentrations with field sampling data used to verify the emission inventory. Our modeling effort focused on the global concentrations in air and seawater from 2010 to 2020.

#### 3.3.1 Modeled OPFR Concentrations in Air and Seawater

**Figure 4:**
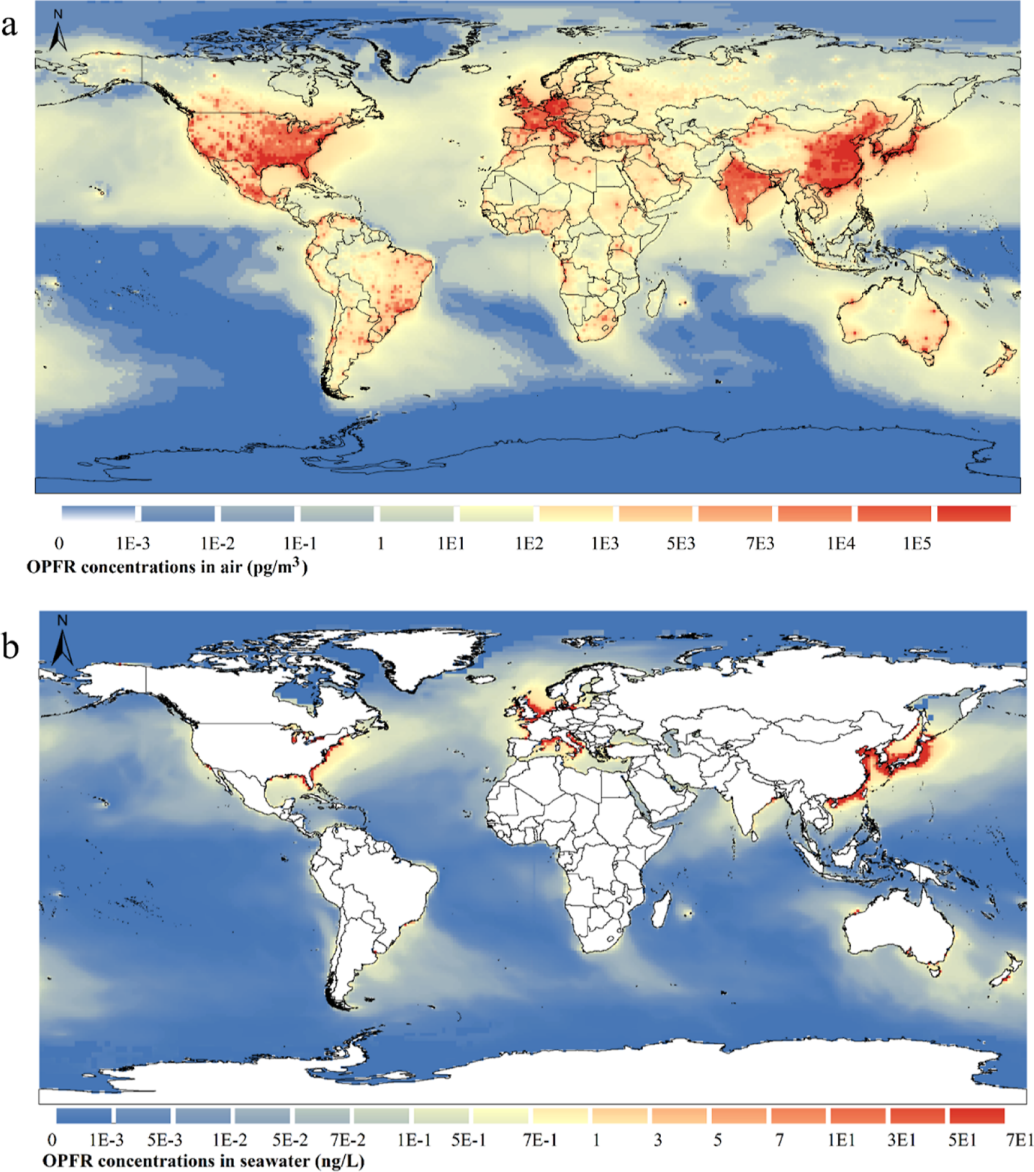
CanMETOP modeled OPFR concentrations in air and seawater in 2020. (a) Air (gas + particle phase, unit: pg/m^3^); (b) seawater (unit: ng/L).

Figure 4a,b shows the spatial distribution of the annually averaged OPFR concentrations in the atmosphere (gas + particle phase) at a height of 1.5 m above the ground surface and seawater in 2020. To be consistent with emissions, higher air concentrations are seen in East Asia, notably in eastern China, Japan, and South Korea, India, West Europe, and the US, again agreeing with the population intensity and industrial activities. The global mean concentrations in air in 2020 were 875.49 (95% CI: 779.07–1000.08) pg/m^3^. We found that, compared with emissions, from a long-term perspective, the effect of the atmospheric transport on OPFR concentrations is most pronounced in those regions with low emissions, such as Mongolia, the country with very low emissions but accounted for a high concentration of 137.66 pg/m^3^, which was mostly transported and diffused from China under the eastern Asian summer monsoon (Figure S4c). Likewise, some African countries were influenced by high-emission countries like Nigeria and the Democratic Republic of the Congo induced by the westerly winds in Western Africa (Figure S4c). Brazil and Mexico, with their high OPFR emissions, also made a substantial contribution to OPFR atmospheric levels in South and Central America, driven by the low-level easterly jet and the trade winds^49^ (Figure S4).

OPFR contamination in seawater is driven by atmospheric transport and subsequent dry/wet deposition, gaseous diffusive exchange between air and water, and surface runoff.^8,45^ The CanMETOP considers the major pathways of OPFR entering seawater, except for surface runoff. Modeled seawater concentrations are illustrated in Figure 4b.^46^ The result indicates that the annual mean concentrations of OPFR over global seawater in 2020 were 0.79 (95% CI: 0.73–0.87) ng/L. Higher concentrations can be seen in the coastal regions of different continents, particularly in the east coast of China, near shore waters around South Korea and Japan, and the western coast of Europe and the east coast of the US, suggesting that land source proximity plays a vital role in the spatial distribution of OPFR in the marine environment across the world.

#### 3.3.2 OPFR Emission Verification

**Figure 5:**
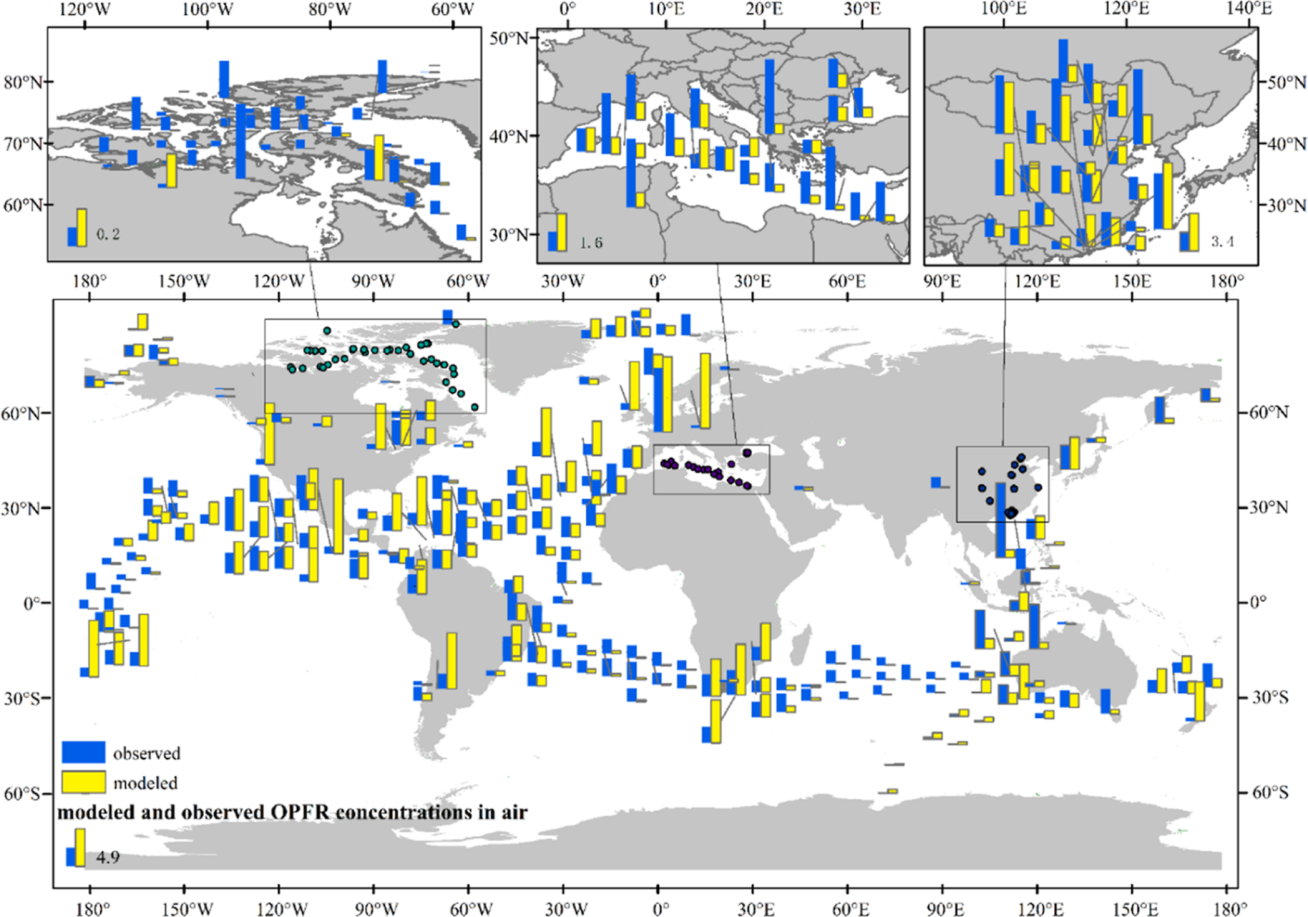
Comparisons between the modeled and observed air OPFR concentrations across global land and ocean.

To ascertain the veracity of the emission inventory, we compared measured OPFR concentrations collected from the literature with the CanMETOP simulated OPFR environmental levels using newly developed OPFR emission inventory as model input. If the simulated concentrations agree with the measured results, we would assume that the emission inventory is sufficiently accurate and reliable. Given that OPFR in the environment are all sourced from anthropogenic activities, their concentrations in the environment emanate solely from emissions from human activities. We compiled 318 measured data records of OPFR in the air and 79 in seawater from literature (Supporting Information 2). The comparison between the observed concentrations at those air sampling sites and the simulated concentration at these sites is depicted in Figure 5. Figure S5 shows observed (blue bar) and simulated (red bar) values in global seawater. Figure S6 shows a correlation diagram between measured and modeled OPFR concentrations in air (Figure S6a) and seawater (Figure S6b), respectively. The linear correlation coefficients for the atmosphere and seawater are *R*^2^ = 0.42 (*p* < 0.001) and 0.72 (*p* < 0.001), respectively. Detailed statistics between modeled and sampled data in air and seawater are presented in Table S7. As seen, the modeled concentrations capture well the measured data with the small deviations and bias. The agreements between modeled and measured concentrations imply that the OPFR emission inventory established in this study is reliable because measured concentrations reflect largely the emission characteristics.^17,37−39,43−45^ However, we also observed underestimated seawater concentrations (Figure 4b), occurring mostly in the Southern Indian Ocean, Southern Atlantic, North Pacific, and near the Arctic Archipelago of Canada. These regions are distant from the land emission sources. Rather, in those regions proximate to the land emission sources, simulated concentrations, either in land or seawater, matched the observations well, without considerable deviations.

In addition to emissions, physicochemical parameters can also disturb marked modeling results. To explore the response of modeled OPFR concentrations to the changes in its physicochemical parameters, we performed sensitivity model simulations by adding a perturbation of ±10% to the physicochemical parameters. Under this condition, resulted air concentrations varied only 3% (Table S4), suggesting that the modeling results were not very sensitive to less considerable changes in physicochemical parameters. The result further confirms that emissions are dominant drivers determining OPFR concentration levels in the different environmental media, particularly in those regions and grid cells proximate to its sources. The good agreements between modeled and sampled data can be considered to be a reasonable criterion measuring the reliability of the emission inventory.

It should be noted that the CanMETOP model only considers major dynamic and thermodynamic processes of POPs’ evolution and transport in the environment but excludes the photochemical reactions because most POPs are persistent with a low activity. However, the photochemical reactions of OPFR in the atmosphere can considerably alter their concentration and persistence. Under intense sunlight, hydroxyl radicals are produced in the atmosphere, which can react with OPFR.^23^ For example, under illuminated conditions, hydroxyl radicals can add to the C–H bond of TCEP to form chlorinated alcohol compound TCEP-1 (with a conversion rate of 4.1%). Similarly, hydroxyl radicals can also attack the C–Cl bond in TCPP to generate TCPP-1 (with a conversion rate of 1.2%).^23^ The addition of oxygen atoms to the OPFR molecules can form oxygen-containing functional groups, such as hydroxyl (−OH) and carbonyl (C=O), which makes the transformation products more polar and may reduce their volatility in nongaseous media, thereby slowing down their degradation rate. These products may increase the persistence of OPFR in the atmosphere and enable them to transport a longer distance in the atmosphere and accumulate at higher concentrations in polar regions with lower light exposure. Since the OPFR in seawater mainly originate from the atmosphere, such a reaction may also occur in seawater (Figure S5), as revealed by elevated levels of OPFR in Arctic seawater and the atmosphere.^47,48^ As a result, the predicted concentrations close to the land emission sources agree better with observed concentrations but are lower than measurements in those regions far away from sources. In fact, the most considerable underestimation of OPFR concentrations in the atmosphere occurs in the polar region. The neglection of atmosphere chemistry (e.g., photochemical reaction) likely yields more rapid degradation of OPFR in air, reducing its lifetime in the environment. In the absence of atmospheric chemistry modules, the CanMETOP model, while possessing a high degree of accuracy, is unable to simulate OPFRs’ photochemical reactions. Further efforts need to be made to understand complex OPFR chemistry in the atmosphere and develop the corresponding modules in the atmospheric transport models. However, considering that the present study aims to develop a global OPFR emission inventory and there are almost no local sources of OPFRs in the polar environment, the photochemical reactions do not impact the emission inventory reported here.

## 4 Implications

The present study compiled and calibrated the global production and consumption volumes of OPFR, which helps us to establish a spatially resolved global emission inventory from 2010 to 2020 on a resolution of 1° × 1°. The new inventory was validated against measured OPFR concentrations in air and seawater across the world. The results revealed a considerable upward trend in global OPFR emissions from 2010 to 2020 and emissions from production as a main source, accounting for more than 55% of the 11 year total. Regionally, those areas with high dense populations and industrialization, such as eastern Asia, western Europe, and North America, contributed the most to the global emissions, where the top ten countries accounted for over 66% of the global total. Given the increasing demand for OPFR, we anticipate that emissions of OPFR will continue to grow in the future. Through comprehensive analysis and simulation with atmospheric transport models, the study provides a holistic perspective on the environmental behavior of global OPFR. We recognize potential significance of atmospheric chemical reaction processes in the global cycling of OPFR. The CanMETOP model failed to capture OPFR evolution in the remote polar region, implying the need of a more comprehensive atmospheric chemistry module that takes photochemical reaction and other chemical processes for OPFR into consideration.

Overall, the present study represents a pioneering endeavor to construct a globally comprehensive and high-precision emission inventory for global OPFR, possessing the unique capability to simulate and validate their environmental concentrations. The incorporation of the model makes the verification of the inventory more scientific and comprehensive while also providing insights into the distribution of environmental concentrations. The inventory will help researchers and policymakers to evaluate the potential risks of OPFR to the environment and human health, develop effective policies and strategies to address global OPFR contamination, monitor the emission trends and sources of OPFR, and forecast their future impacts.

## Supporting Information Available

Flowchart of OPFR emission inventories, description for CanMETOP model, sensitivity analysis, global nighttime light in 2020, OPFR concentrations in air at an altitude of 1200 m in 2020, frequency distribution of OPFR emission from various emission categories, and physicochemical properties of OPFR used in CanMETOP model simulation (PDF)
Data of production and consumption volume of OPFR (Supplementary Data 1) (XLSX)
Data of comparison between the observed and modeled concentrations (Supplementary Data 2) (XLSX)
