# Supplementary material for: Global Gridded Emission Inventory of Organophosphate Flame Retardants from 2010 to 2020
Source: Environ Sci Technol. 2024 Sep 9;58(38):17070–80. doi: 10.1021/acs.est.4c06504 (PMC11428127; doi:10.1021/acs.est.4c06504)
Supplement: Supplementary file 1 — es4c06504_si_001.pdf [file es4c06504_si_001.pdf]

Supporting Information

for

Global Gridded Emission Inventory of Organophosphate Flame Retardants from 2010 to 2020

Haibo Ma†, Chao Wang†, Huabing Suo†, Yandi Huang†, Yuanhui Huo†, Gang Yang†, Yu Yan†, Tao Huang†, Hong Gao†*, Jianmin Ma‡, Zhiyong Xie□**

Affiliations:

† Key Laboratory for Environmental Pollution Prediction and Control, Gansu Province, College of Earth and Environmental Sciences, Lanzhou University, Lanzhou 730000, P. R. China

‡ Laboratory for Earth Surface Processes, College of Urban and Environmental Sciences, Peking University, Beijing 100871, P. R. China

□ Helmholtz-Zentrum Hereon, Institute of Coastal Environmental Chemistry, Geesthacht, 21502, Germany

Corresponding author:

*Hong Gao, College of Earth and Environmental Sciences, Lanzhou University, Lanzhou 730000, P. R. China

Email: honggao@lzu.edu.cn

**Zhiyong Xie, Helmholtz-Zentrum Hereon, Institute of Coastal Environmental Chemistry, Geesthacht, 21502, Germany

Email: zhiyong.xie@hereon.de

Number of Pages: 14

Number of Texts: 2

Number of Figures: 6

Number of Tables: 7

The Supporting Information file includes the following sections:

Supporting Text S1-S2 ....S5

Supporting Figures S1-S6 ....S8

Supporting Tables S1-S7 ....S12

Supporting References S14

Text S1. CanMETOP model

CanMETOP is an advanced three-dimensional atmospheric transport model that employs an advection-diffusion equation methodology, accounting for the fundamental dynamic and thermodynamic processes present within the atmosphere and the atmospheric boundary layer. This model combines physical and chemical properties of pollutants, emission data, meteorological data (such as temperature, wind speed, precipitation, and atmospheric pressure), and surface characteristic data. CanMETOP is coupled with modules simulating POPs (persistent organic pollutants) cycling via water–air exchange, soil, and sediment, which enables quantitative prediction of the fate and transport of POPs in various environmental media and their spatiotemporal variations in the atmosphere. CanMETOP was designed to simulate environmental fate in multiple environmental media and widely used in the study of source-sink relationships, multiple environmental fate, and climate change effect of POPs on reginal and global scales [1-3]. The model is numerically solved utilizing a modified Bott's algorithm[4] to address the horizontal advection term and a central-difference method to elucidate the horizontal diffusion term. In the vertical direction, finite difference methodologies are employed to approximate the vertical advection and diffusion terms of the atmospheric diffusion equation. In the current version of the model, a semi-implicit forward method is applied to solve the vertical advection and a semi-implicit Crank-Nicholson scheme is utilized to solve the vertical diffusion term.

The CanMETOP considers various transport and removal pathways for POPs, including atmospheric advection, dry and wet deposition, scavenging through precipitation, diffusion, and soil leaching and degradation. The soil-air exchange model uses a dynamic, three soil layer, fugacity-based model. The presented conceptual model is utilized to identify the degree of dis-equilibrium and the magnitude of the soil-to-air transfer. Within the soil compartment, which is assumed to contain a single soil type, the soil is represented by three distinct layers. Each layer is characterized by a combination of chemical loss mechanisms, including volatilization, leaching, degradation, and diffusion. The transport of pesticide by upward bulk water flow (wicking) is not considered in this model, because this is not important for POPs. Removal of a chemical by reaction in air via OH radical is also not included in the model, because the residence time for the model grid cell (a few hours) is very small compared to typical half-times for atmospheric reaction processes [5].

The water-air exchange process is mathematically simulated using a two-film model[6], which recognizes the significant role of molecular diffusion in limiting the rate of net mass transfer across the air-water interface. This mathematical model employs a simplified conceptualization of the transfer process, where the flow of material is restricted by theoretical considerations of material transport through thin, two-dimensional films of air and water adjacent to the fluid-solid interface.

CanMETOP is capable of simulating soil concentrations. However, soil pollution of OPFRs is caused mainly wastewater discharge and atmospheric deposition [7]. Since the emission inventory developed in this study focuses on the atmospheric emissions process only, the wastewater discharge process was not considered. In the absence of wastewater discharge, the atmospheric deposition becomes a major pathway for soil pollution. As a result, the modeled OPFR concentrations in soil were very low. In addition, field measured soil samples are very rare, so that the sampled soli concentration data could not be used to verify the emission inventory from a statistics perspective.

Text S2. CanMETOP model sensitivity analysis

The CanMETOP model applied in validating OPFR emission inventory comprises intricate dynamics and physical processes such as dry and wet deposition, precipitation scavenging, horizontal and vertical advection, and turbulent diffusion. A first-order error propagation method [8] is employed to assess the uncertainty (Cf_{out}) in modeled concentrations predicted by the CanMETOP model. This method evaluates the uncertainty of the simulated concentrations by propagating the uncertainty of the model input parameters (Cf_i), defined by

$$Cf_{out} = exp\sqrt{\sum_i (lnCf_i)^2 \times S_i^2} \quad \text{(S1)}$$

where Cf_{out} and Cf_i represent the confidence factors (Cf) of input and output variables in the CanMETOP following a log-normal distribution within a 95% confidence interval. S_i denotes the relative sensitivity (S) of the model output to changes in input parameter *I*, given by

$$S = (\frac{\Delta O}{O})/(\frac{\Delta I}{I}) \quad \text{(S2)}$$

where ΔI and ΔO are the changes (differences) in input parameters and output values of the model responding to input values. The average sensitivity of the model to alterations in each input parameter is determined by increasing or decreasing each input parameter by 10%.

Modeled concentration of simulated gridded concentrations within the 95% confidence interval (CI) is defined by

$$95\%CI\colon\left\{\frac{u}{Cf_{out}} < x < u \cdot Cf_{out}\right\} \tag{S3}$$

where Cf_{out} represents the extent to which x may deviate from the median value u. The details of the input parameters in the model are presented in Table S4. Uncertainty and sensitivity of meteorological variables in CanMETOP modeling were not examined because confidence factors aren't explicitly defined as a single, standard metric in an atmospheric transport model, and the concept of "confidence" in CanMETOP is more nuanced and multifaceted. Uncertainty in the initial conditions, model parameters, and physical processes propagates associated with meteorology make it difficult to quantify a single "confidence" value. For example, confidence in input meteorological variables can vary drastically depending on location and time.

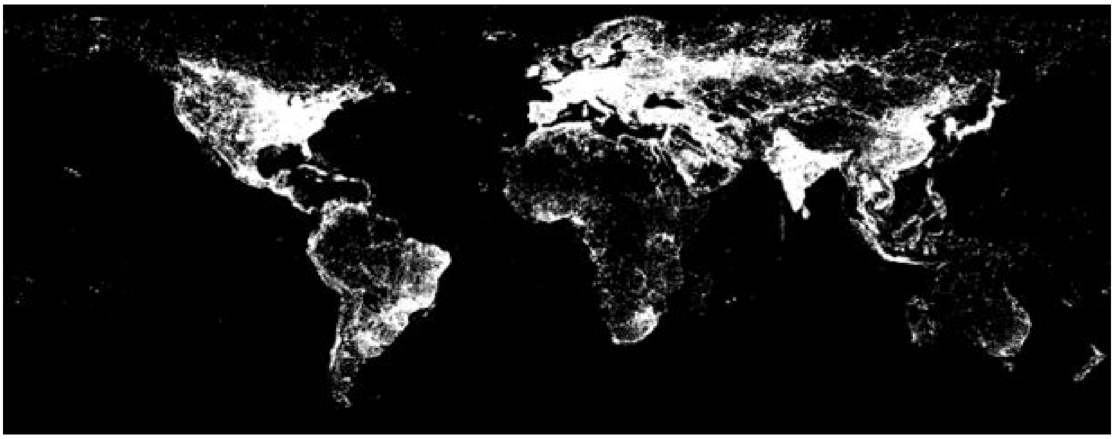

Figure S1. Global nighttime light in 2020.

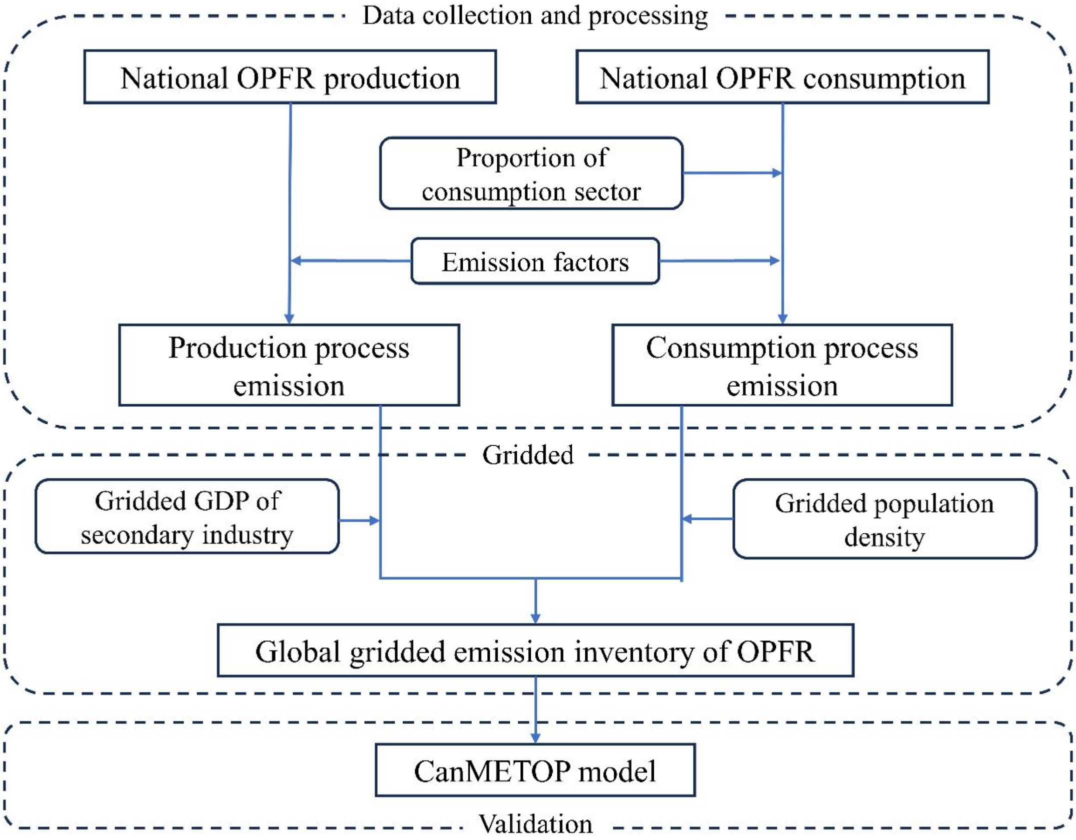

Figure S2. Flowchart showing the steps and procedures for establishing global gridded OPFR emission inventories.

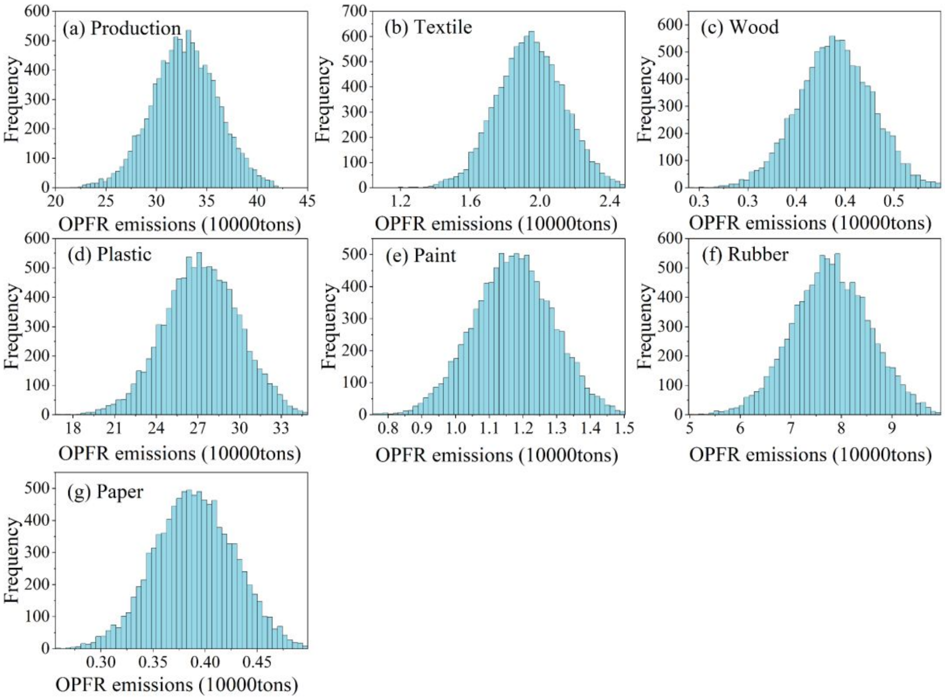

Figure S3. Frequency distribution of OPFR emission from various emission sectors.

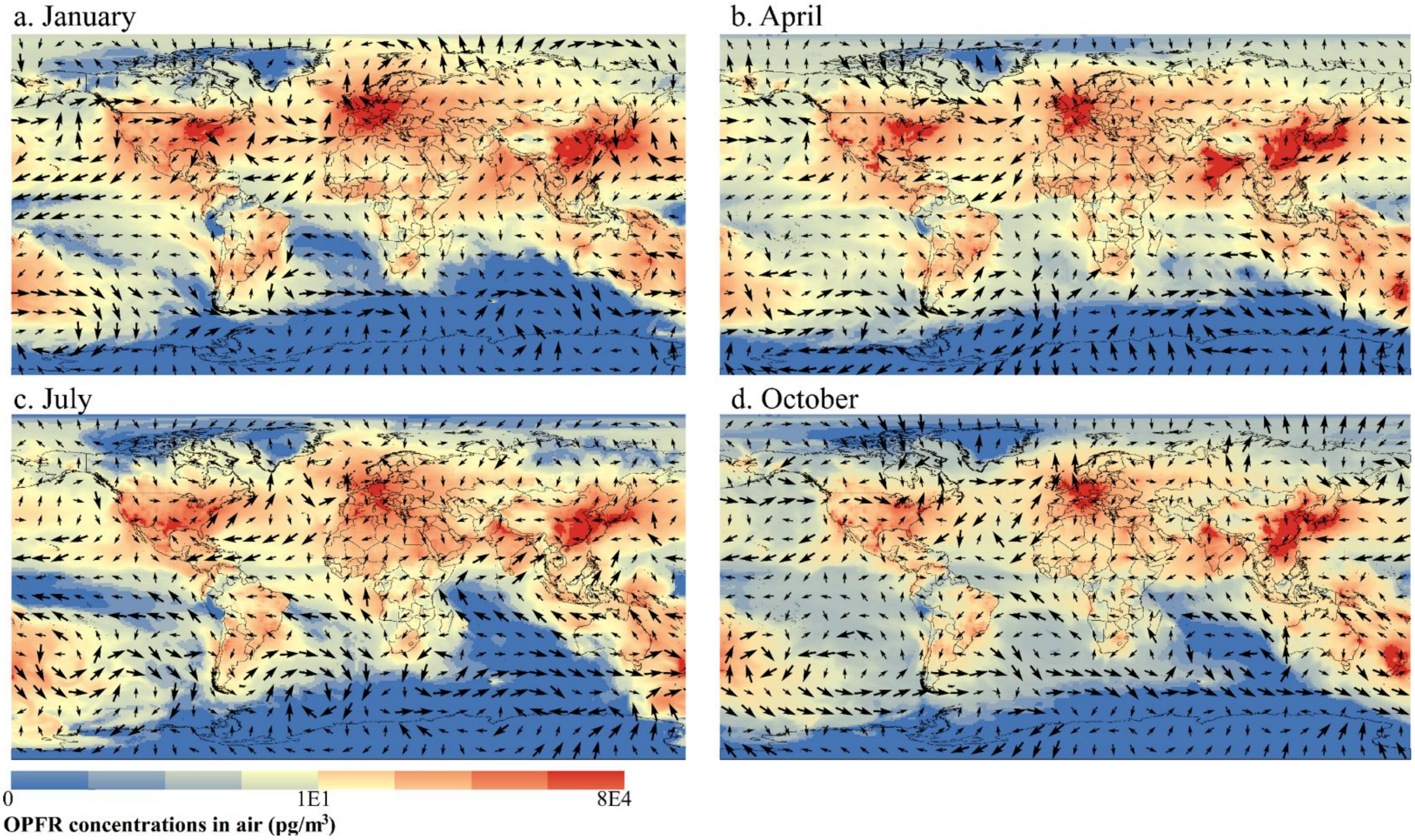

Figure S4. CanMETOP modeled monthly mean OPFR concentrations in air at the altitude of 1200 meters in 2020. a. January, b. April, c. July, and d. October. Black arrows indicate wind vectors.

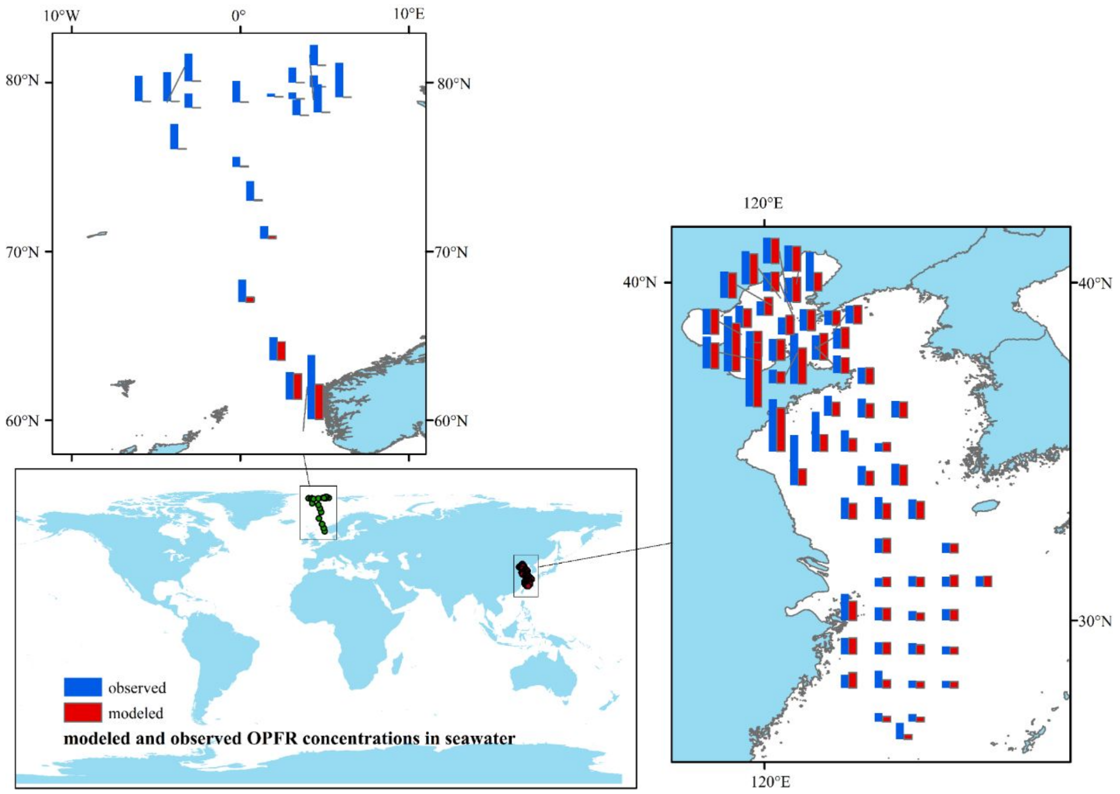

Figure S5. Comparison of modeled and sampled concentrations of OPFR in seawater.

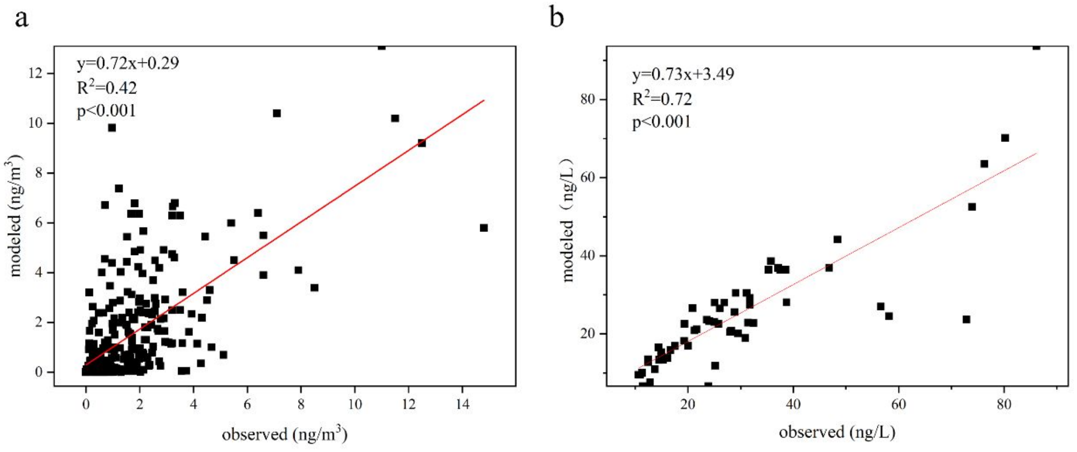

Figure S6. Comparison between modeled and observed concentrations of OPFRs in air (a) and seawater (b), where R^2 is correlation coefficients between modeled and measured concentrations.

Table S1. Atmospheric emission factors of OPFR in production and consumption sectors (%) [9]

Emission sources		Processes		Scenarios	Emission factor
Production		Handling of raw materials			0
		Synthesis			0.005
		Conversion			0.25
Use					
	Plastics	Handling of raw materials			0
		Mixing		Low scenario	0.001
				Middle scenario	0.005
				High scenario	0.025
		Conversion	Closed process （80%）	Low scenario	0.001
				Middle scenario	0.005
				High scenario	0.025
			Partial opening process（20%）	Low scenario	0.003
				Middle scenario	0.015
				High scenario	0.075
		Finished product	Indoor		0.05
			Outdoor		0.05
	Rubber				0.1
	Textiles				1.9
	Coatings				0.23
	Paper				0.26
	Wood				0.0116

Table S2. Names, abbreviations, formulae, and physical-chemical properties of OPFRs adopted in the present study [10-12].

Acronym	Name	CAS	Formula	Solubility (mg/L) 25˚C	Log K_{OW}	log K_{OC}	log K_{OA}	Vapor pressure (mmHg) （25 ˚C）	Henry's law constant (atm/m^3/mol)（25˚C）	Half-life (medium)	BCF
TEP	Triethyl phosphate	78-40-0	$C_6H_{15}O_4P$	5.0×10^5	0.8	1.86	6.63	0.29	3.5×10^{-6}	-	3.2
TPP	Tripropyl phosphate	513-08-6	$C_9H_{21}O_4P$	8.3×10^2	2.67		6.42	2.9×10^{-2}	8.2×10^{-6}	-	0.9
TIBP	Tri-iso-butyl phosphate	126-71-6	$C_{12}H_{27}O_4P$	3.7	3.6		7.48	1.3×10^{-2}	1.1×10^{-4}	4.3 h (atmosphere)	19.5
TNBP	Tri-n-butyl phosphate	126-73-8	$C_{12}H_{27}O_4P$	2.8×10^2	4	2.83	9.21	1.1×10^{-3}	1.5×10^{-7}	<1 h (atmosphere)	39.8
TCEP	Tris(2-chloroethyl) phosphate	115-96-8	$C_6H_{12}Cl_3O_4P$	7.0×10^3	1.44	3.05	7.42	1.1×10^{-4}	3.3×10^{-6}	17.5 h (atmosphere)	0.4

TCPP	Tris(2-chloroisopropyl) phosphate	13674-84-5	$C_9H_{18}Cl_3O_4P$	1.6×10^{3}	2.59	2.71	8.2	1.9×10^{-6}	6.0×10^{-8}	8.6 h (atmosphere)	3.3
TBEP	Tris(2-butoxyethyl) phosphate	78-51-3	$C_{18}H_{39}O_7P$	1.2×10^{3}	3.65	4.83	13	2.1×10^{-7}	3.3×10^{-11}	3 h (atmosphere)	25.6
TDCPP	Tris(1,3-dichloroisopropyl) phosphate	13674-87-8	$C_9H_{15}Cl_6O_4P$	1.5	3.8	2.35	10.6	7.4×10^{-8}	2.6×10^{-9}	21.3 h (atmosphere)	21.4
TPP	Triphenyl phosphate	115-86-6	$C_{18}H_{15}O_4P$	1.9	4.59	3.72	8.45	1.2×10^{-6}	3.3×10^{-6}	50-60 d (pond hydrosoil)	113
EHDPP	2-Ethylhexyl diphenyl phosphate	1241-94-7	$C_{20}H_{27}O_4P$	1.9	5.37	4.21	8.92	6.5×10^{-7}	2.5×10^{-7}	-	855
TEHP	Tris(2-ethylhexyl) phosphate	78-42-2	$C_{24}H_{51}O_4P$	0.6	9.49	6.87	14.9	2.0×10^{-6}	9.6×10^{-5}	-	3.2
TMPP	Tris(methylphenyl) phosphate	78-32-0	$C_{21}H_{21}O_4P$	0.36	5.11	4.35	5.88	1.8×10^{-7}	9.2×10^{-7}	7.5 h (sewage sludge)	2543

Table S3. Physicochemical properties of OPFR used in CanMETOP model simulation [13].

Parameters	Value	Parameters	Value
Molecular mass（g/mol）	285.49	Degradation rate in soil（S^{-1}）	6.10×10^{-9}
Molar volume（cm^3/mol）	205.39	Liquid vapor pressure（Pa）	1.10×10^{-4}
Melting point（˚C）	26.70	Solubility (g/m^3)	9.92×10^{2}
Entropy of fusion (J/mol K)	74.50	log K_{ow}（25˚C）	3.98
Degradation rate in air（S^{-1}）	1.98×10^{-7}	log K_{oa}（25˚C）	8.38

Table S4. Input confidence factors (C*f*) for physicochemical parameters [14, 15].

Physicochemical parameters	Value	Confidence factors (C*f*)
Molecular mass (g/mol)	314.04	1
Molar volume (cm^3/mol)	225.93	1
Melting point (℃)	29.37	1
Log K_{oa} (25℃)	8.29	1.1
Vapor pressure (mmHg)	1.21×10^{-4}	1.5
Log K_{ow} (25℃)	4.38	1.5
Henrys Law Constant (25℃) (atm-m^3/mol)	6.95	1.5

Degradation rate in air ($cm^3 \cdot molecule^{-1} \cdot h^{-1}$)	2.18×10^{-7}	2
Degradation rate in soil (S^{-1})	6.71×10^{-9}	2
Solubility (g/m^3)	1.09×10^{3}	1.5

Table S5. Coefficient of variation (CV, %) for various emission sources incorporated in uncertainty analysis using Monte Carlo model.

Emission categories	CV/%	
	Use ratio	Emission factor
Production	-	43.8
Application	-	-
Plastics	10	17.9
Rubber	10	19.9
Textiles	10	32.8
Coatings	10	29.8
Paper	10	29.5
Wood	10	29.9

Table S6. Annual OPFR atmospheric emissions in the globe from seven sources summed from 2010 to 2020 (tons/year).

Years	Emission categories							
	Plastic	Rubber	Textile	Coating	Wood	Paper	Production	Total
2010	288.50	74.93	355.94	25.85	9.74	0.43	928.96	1684.37
2011	296.43	76.99	365.73	26.56	10.01	0.44	952.42	1728.60
2012	304.41	79.06	375.57	27.27	10.27	0.45	981.21	1778.28
2013	312.63	81.20	385.72	28.015	10.55	0.47	996.54	1815.14
2014	321.71	83.56	396.92	28.82	10.86	0.48	1020	1862.38
2015	331.24	86.03	408.67	29.68	11.18	0.49	1047.54	1914.86
2016	341.99	88.83	421.94	30.64	11.54	0.51	1081.2	1976.67
2017	350.01	90.91	431.82	31.36	11.81	0.52	1110.27	2026.71
2018	359.57	93.39	443.63	32.22	12.14	0.54	1173.53	2115.05
2019	358.84	93.20	442.73	32.15	12.11	0.54	1243.34	2182.94
2020	364.10	94.57	449.22	32.62	12.29	0.54	1285.98	2239.37

Table S7. Statistics of model evaluation results at the air and water sampling sites. [9]

Statistical measure	Formula	Air	Water
Fraction of model values within a factor of two of measured OPFR	$\mathrm{FA2} = \frac{\sum_{i=1}^{N}(0.5 \leq M_i/O_i \leq 2.0)}{N} \times 100\%$	40%	65%
Fraction of model values within a factor of five of measured OPFR	$\mathrm{FA5} = \frac{\sum_{i=1}^{N}(0.2 \leq M_i/O_i \leq 5.0)}{N} \times 100\%$	76%	68%

Fractional Bias	$\mathrm{FB} = 2(\frac{\overline{M} - \overline{O}}{\overline{M} + \overline{O}})$	-0.09	0.19
Standard Deviation	$\mathrm{STD} = \sqrt{\frac{\sum_{i=1}^{N} (x_i - \bar{x})^2}{N - 1}}$	1.87	17.4
Bias	$\mathrm{BIAS} = \frac{1}{N} \sum_{i=1}^{N} (M_i - O_i)$	-0.13	-4.11
Fraction	$\mathrm{FRA} = \frac{\sum_{i=1}^{N} (M_i - O_i)}{\sum_{i=1}^{N} O_i}$	-0.09	-0.18
Mean Absolute Difference	$\mathrm{MAD} = \frac{1}{N} \sum_{i=1}^{N} \lvert M_i - O_i \rvert$	1.05	4.87
Normalized Mean Absolute Difference	$\mathrm{NMAD} = \frac{\sum_{i=1}^{N} \lvert M_i - O_i \rvert}{\sum_{i=1}^{N} O_i}$	0.70	0.21

References

1. Ma, J., S. Daggupaty, T. Harner, and Y. Li, *Impacts of Lindane Usage in the Canadian Prairies on the Great Lakes Ecosystem. 1. Coupled Atmospheric Transport Model and Modeled Concentrations in Air and Soil. Environmental Science & Technology*, **2003**. *37*(17): p. 3774-3781.10.1021/es034160x
2. Huang, T., W. Jiang, Z. Ling, Y. Zhao, H. Gao, and J. Ma, *Trend of cancer risk of Chinese inhabitants to dioxins due to changes in dietary patterns: 1980–2009. Scientific Reports*, **2016**. *6*(1): p. 21997.10.1038/srep21997
3. Tian, C., J. Ma, L. Liu, H. Jia, D. Xu, and Y.-F. Li, *A modeling assessment of association between East Asian summer monsoon and fate/outflow of α-HCH in Northeast Asia. Atmospheric Environment*, **2009**. *43*(25): p. 3891-3901.https://doi.org/10.1016/j.atmosenv.2009.04.056
4. Walcek, C.J. and N.M. Aleksic, *A simple but accurate mass conservative, peak-preserving, mixing ratio bounded advection algorithm with FORTRAN code. Atmospheric Environment*, **1998**. *32*(22): p. 3863-3880.https://doi.org/10.1016/S1352-2310(98)00099-5
5. Harner, T., T.F. Bidleman, L.M.M. Jantunen, and D. Mackay, *Soil—air exchange model of persistent pesticides in the United States cotton belt. Environmental Toxicology and Chemistry*, **2001**. *20*(7): p. 1612-1621.https://doi.org/10.1002/etc.5620200728
6. Liss, P.S. and P.G. Slater, *Flux of Gases across the Air-Sea Interface. Nature*, **1974**. *247*(5438): p. 181-184.10.1038/247181a0
7. Wang, Y., Z. Li, F. Tan, Y. Xu, H. Zhao, and J. Chen, *Occurrence and air-soil exchange of organophosphate flame retardants in the air and soil of Dalian, China. Environmental Pollution*, **2020**. *265*: p. 114850.https://doi.org/10.1016/j.envpol.2020.114850
8. Huang, T., Z. Ling, J. Ma, R.W. Macdonald, H. Gao, S. Tao, C. Tian, S. Song, W. Jiang, L. Chen, K. Chen, Z. Xie, Y. Zhao, L. Zhao, C. Gu, and X. Mao, *Human exposure to polychlorinated biphenyls embodied in global fish trade. Nature Food*, **2020**. *1*(5): p. 292-300.10.1038/s43016-020-0066-1
9. He, J., Z. Wang, L. Zhao, H. Ma, J. Huang, H. Li, X. Mao, T. Huang, H. Gao, and J. Ma, *Gridded emission inventory of organophosphorus flame retardants in China and inventory validation. Environmental Pollution*, **2021**. *290*: p. 118071.https://doi.org/10.1016/j.envpol.2021.118071
10. van der Veen, I. and J. de Boer, *Phosphorus flame retardants: Properties, production, environmental occurrence, toxicity and analysis. Chemosphere*, **2012**. *88*(10): p. 1119-1153.https://doi.org/10.1016/j.chemosphere.2012.03.067
11. Wan, W., S. Zhang, H. Huang, and T. Wu, *Occurrence and distribution of organophosphorus esters in soils and wheat plants in a plastic waste treatment area in China. Environmental Pollution*, **2016**. *214*: p. 349-353.https://doi.org/10.1016/j.envpol.2016.04.038
12. Wei, G.-L., D.-Q. Li, M.-N. Zhuo, Y.-S. Liao, Z.-Y. Xie, T.-L. Guo, J.-J. Li, S.-Y. Zhang, and Z.-Q. Liang, *Organophosphorus flame retardants and plasticizers: Sources, occurrence, toxicity and human exposure. Environmental Pollution*, **2015**. *196*: p. 29-

46.https://doi.org/10.1016/j.envpol.2014.09.012

13. Pantelaki, I. and D. Voutsa, *Organophosphate flame retardants (OPFRs): A review on analytical methods and occurrence in wastewater and aquatic environment. Science of The Total Environment*, **2019**. *649*: p. 247-263.https://doi.org/10.1016/j.scitotenv.2018.08.286
14. MacLeod, M., A.J. Fraser, and D. Mackay, *Evaluating and expressing the propagation of uncertainty in chemical fate and bioaccumulation models. Environmental Toxicology and Chemistry*, **2002**. *21*(4): p. 700-709.https://doi.org/10.1002/etc.5620210403
15. Li, H., Z. Wang, J. He, N. Zhang, X. Mao, J. Ma, H. Gao, Z. Yang, and H. Ma, *Deca-BDE emissions, validation, and environmental fate in China. Journal of Hazardous Materials*, **2023**. *459*: p. 132223.https://doi.org/10.1016/j.jhazmat.2023.132223
